# Prevalence and determinants of intimate partner violence against mothers of children under-five years in Central Malawi

**DOI:** 10.1186/s12889-020-09910-z

**Published:** 2020-12-02

**Authors:** Emmanuel Chilanga, Delphine Collin-Vezina, Mohammad Nuruzzaman Khan, Liam Riley

**Affiliations:** 1 Department of Geography, University of Livingstonia, Livingstonia, Malawi; 2 Centre for Research on Children and Families, School of Social Work, McGill University, Montreal, Canada; 3 Faculty of Social Work, University of Manitoba, Winnipeg, Manitoba Canada; 4 Balsillie School of International Affairs, Wilfrid Laurier University, Waterloo, Canada

**Keywords:** Intimate partner violence, Mothers, Prevalence, Risk factors, Malawi

## Abstract

**Background:**

Intimate partner violence (IPV) against women is a global human rights violation and a public health problem. The phenomenon is linked to adverse health effects for women and children. Mothers of young children in Malawi can be particularly at risk because of gender-based power imbalances. The objectives of this study were to examine the prevalence and the risk factors of IPV against mothers of children under-five years of age in rural Malawi.

**Methods:**

A multistage, cross-sectional study design was used. A sample of 538 mothers of young children was randomly selected from postnatal clinics in Dowa district. The WHO’s Violence against women screening instrument was used to collect data. Logistic regressions were used to determine risk factors that were associated with IPV against mothers.

**Results:**

Overall prevalence of all four forms of IPV against mothers of under-five children was 60.2%. The prevalence of IPV controlling behavior, psychological, physical, and sexual violence were 74.7, 49.4, 43.7 and 73.2% respectively. In multivariate analyses, mothers whose partners had extra marital affairs were more likely to experience controlling behavior (AOR: 4.97, 95% CI: 2.59–8.55, *P* = 0.001), psychological (AOR: 2.14, 95% CI: 1.486–3.472, *P* = 0.001) and physical (AOR: 2.29, 95% CI: 1.48–3.94, P = 0.001) violence than mothers whose partners did not have extra marital affairs. Mothers whose partners consume alcohol were more likely to experience sexual violence (AOR: 2.00, 95% CI: 1.17–3.41, *P* = 0.001) than mothers whose partners did not drink. Finally, mothers who spent more than 30 min drawing water were at greater risk of experiencing IPV than mothers who spent less than 30 min.

**Conclusion:**

This study found a significantly higher prevalence of IPV against mothers of under-five children in rural Malawi compared to women in the general population. Micro and macro-level programs aimed at mitigating the partners’ potential risk behaviors identified in this study are suggested. Public health programs that support increased household access to safe water are also recommended to help undermine IPV against mothers.

## Background

Intimate partner violence (IPV) against women is a pervasive public health problem and a human rights violation [1]. The term IPV refers to any form of physical, sexual, psychological (emotional), and economic abuse that occurs between former, current, or dating partners. Globally, about 35% of women have experienced either sexual and/or physical violence perpetrated by an intimate partner [2]. The burden of IPV against women in Sub Saharan Africa (SSA) is high. Approximately 45.6% of the women in this African region have experienced at least one form of IPV in their lifetime [3]. In Malawi, a 2016 demographic survey indicated that 42% of women experienced at least one form of IPV at some point in their lives [4].

In SSA, IPV has been linked to numerous negative health outcomes for women [5]. For instance, a study in Malawi found that IPV against women was a risk factor for still-birth, abortion, and premature delivery [6]. In Ghana, IPV against women was associated with physical injuries, increased risk of contracting HIV, and depression [7]. Studies in SSA have also established that IPV against mothers has a negative effect on child well-being. In a longitudinal study conducted in Tanzania, maternal exposure to sexual and physical IPV was associated with child stunting and delayed motor skills and cognitive development [8]. Another study in Tanzania found that IPV against mothers was a risk factor for child morbidity [9]. Given these serious effects on maternal and child health, continued research into IPV against women in SSA is critically important. In particular, the engagement of the fields of public health and social work is needed to build knowledge and effective responses in this area of knowledge.

To address IPV and related human rights violation against women, the African Union in 2010 launched the African Women Decade (AWD) [10]. The goal was to accelerate gender equality and empowerment of women through a top down and bottom up framework with particular focus on grassroots participation. The current study provide localized insights to the implementation of AWD objectives with particular focus on three themes. These are: (1) Women’s health, maternal mortality and HIV&AIDS, (2) Peace and security and violence against women, and (3) Mentoring men and women to be champions of gender equality and women’s empowerment [10]. The study provides empirical evidence regarding the burden and risk factors of IPV to be used to advocate for gender equality and women empowerment in rural Malawi.

Conceptually, ecological theory is used to understand the determinants of IPV against women that operate at different levels [11]. The theory suggest that individual characteristics of violent behavior are not exclusively determined by pathological factors. Rather, they are nested within the broader context of culture and policies that reinforce specific values and behaviors. By using ecological lenses, IPV research in SSA has documented that at personal level, men and women who experienced abuse in childhood, consume alcohol, accept male dominance, and have lower educational levels are more likely to perpetrate or experience IPV respectively [3, 12]. At the community and macro levels, poverty, hegemonic masculine norms, and weak law enforcement have been identified as significant risk factors for violence against women [3].

A growing body of research in developing countries including Malawi has examined the prevalence of IPV by focusing on samples of women in the general population, during pregnancy [13], during the postpartum period [14], and among girls and young women. These studies have shown that women’s experience of IPV is a predisposing risk factor for maternal and child morbidity. A pioneering study in India has suggested that children of mothers who experienced IPV in the past 12 months prior to the study were 1.37, and 1.65 times more likely to suffer from acute respiratory infection (ARI), and diarrhea respectively [15]. Lately, a study in South Asia has confirmed that IPV against mothers increases the health risk of ARI, fever, and diarrhea among under-five children [16]. In SSA, a study conducted in Tanzania has found that children who were in post-natal and early childhood periods were at an increased risk of morbidity when their mothers were exposed to IPV [9].

In Malawi, morbidity in children under the age of five is a significant public health problem that exacerbates child mortality [17]. Although various interventions such as universal child health care coverage and farm subsidies have been implemented, child morbidity is still unacceptably high [4]. Regarding undernutrition, current statistics have suggested that 37% of the under five-years-old children are too short for their age (stunting), 3% are too thin for their height (wasting), and 12% are too thin for their age (underweight) [4]. In addition, a high proportion of Malawian children are still suffering from infectious diseases that can be prevented through basic hygiene practices, such as provisioning of safe complementary foods and liquids that are needed along with the breast milk [17].

UNICEF’s care for nutrition framework underscores that poor care for women significantly diminishes their capacity for taking care of their children’s health and emotional needs. A literature review by Engle et al. (2000) [18] has highlighted an array of care practices for women that facilitate proper child nutrition and general well-being [18]. These include reducing their workload and desisting from abusing pregnant and lactating women. Some studies in SSA have shown that protecting women from IPV has the potential to enhance their role as the primary caregiver for children. For example, a study in Ghana found that women who received both psychological and physical support from their partners were more likely to provide their children with high dietary diversity, completed immunizations, and adequate personal hygiene practices than mothers who were ill-treated by their partners [19]. Two explanations have been made for the association between IPV against women and child poor health outcomes. It is argued that IPV against mothers undermines their autonomy to implement better childcare practices due to their partners’ controlling behavior. Some studies have postulated that IPV and lack of social support exacerbate maternal stress which can, in the long run, reduce a mother’s capacity to provide care for her child.

Limited research has focused on the prevalence and determinants of IPV against mothers of under-five year old children in rural areas of Malawi. This gap in the research literature merits attention given that IPV against mothers has been shown to increase poor health outcomes in both mothers and their children in developing countries and in the study area. To date, no research has specifically examined the prevalence and risk factors of IPV against mothers of children under five years of age perpetrated by the current or recent partner in rural areas of Dowa district in central Malawi. The current study addresses this gap in order to bolster AWD goals of promoting gender equality and empowerment of women specifically in remote areas of Malawi.

### Objective

The goal of the study was to determine the burden and risk factors of IPV among mothers of under-five children living in rural areas of Dowa district in Malawi. The research project is grounded in UNICEF’s “care for nutrition” conceptual framework that is used to understand immediate and underlying risk factors that aggravate maternal and child poor health outcomes in developing countries [20].

## Methods

### Study setting

**Fig. 1:**
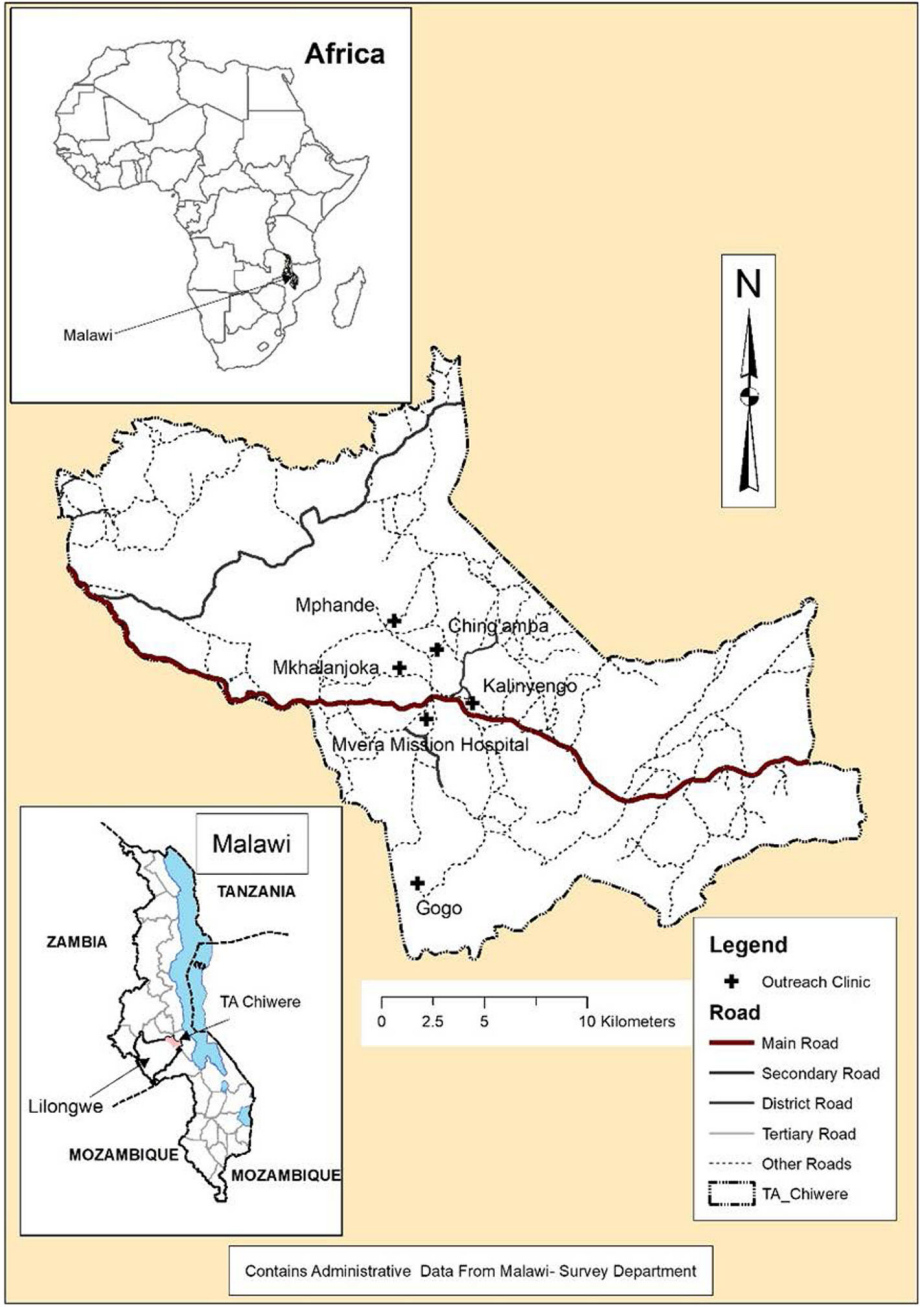
Location of the study area. Source: First author

The study was conducted in Malawi, a small landlocked country of about 118,484 km^2^ in Southern Africa, bordered by Tanzania to the north, Mozambique to the South east, and Zambia to the west. The current population of Malawi is about 17,563,749 people [4]. Eighty-five percent of the population depends on agriculture for their livelihood [4]. This research was specifically conducted in rural agricultural areas approximately five to ten kilometers around Mvera mission hospital in Dowa district (Fig. 1) between the months of May and September 2018. Dowa district was purposively selected in central Malawi because of media reports that show increased cases of homicide that emanated from domestic violence and marital problems [21].

### Study sample

This descriptive cross-sectional study used a multi-stage cluster sampling technique to select representative research participants [22]. Among the eight-outreach clinics under Mvera hospital, six were randomly selected. The selected outreach clinics were Mkhalanjoka, Gogo, Mvera, Kalinyengo, Mphande, and Ching’amba. During the time of the study, Mvera hospital was serving a population of 27,719 people. Out of the total population, there were 4820 under-five year old child/mother dyads that were clients of postnatal health services in the selected six-outreach clinics. A Raosoft online software program was used to calculate a sample size as proposed by McCrum-Gardner, 2010 [23]. The margin of error was set at 5%, with 95% confidence level, and a response distribution of 50%. A systematic sampling strategy was used to select a sample of 538 mothers with under-five children from the total population of 4820 that were recorded in postnatal registers. A first name of a mother was randomly picked and subsequently picked every 9th child-mother dyad.

### Participant recruitment

Selected mothers were contacted through their antenatal clinic when attending their regular monthly health assessment with their youngest under-five child. In a private clinic consultation room, the health worker asked the mother if she would be interested in taking part in this study. If the mother consented, a health worker research assistant administered the questionnaire orally in the consultation room. In this way, the confidentiality of mothers were protected as there was surety that no one present was aware that the mother took part in this study.

### Measures

#### Outcome variables

The primary outcome variable for this study was prevalence of IPV against mothers of children under five years of age perpetrated by the current or most recent partner. We acknowledge that the definition of IPV is a multidimensional concept [24] but in this study the term was operationalized by focusing on IPV against mothers of under five year old children that was perpetrated by the current or recent male sexual partner. The term current or recent partner was defined as a mother’s sexual partner who was the father of the under-five child that the mother was nursing. They could be still in a sexual relationship or separated at the time of the interview. Mother’s exposure to IPV was screened using a WHO violence against women multi-country questionnaire instrument on women’s health and life experiences that had been validated and used in Malawi [12, 25].

The questionnaire contains 18 items that make up four sub-scales measuring different forms of IPV: physical, emotional, controlling behavior, and sexual abuse. Maternal exposure to each [26] form of IPV was defined as the mother giving a positive answer to any one of the questions within each subscale. For instance, if a respondent answered “Yes” to any of the six items under controlling behavior, it was counted that such a mother was a victim of controlling violence. The study conformed to the standard IPV exposure screening instrument that was used in prior studies.

Mothers were considered exposed to psychological abuse if reported ever been belittled, insulted, hurt or scared by the current or recent partner. A mother was considered a victim of physical violence if affirmed encountered one of the following situations: was ever punched, slapped, kicked, pushed, choked, or threatened with a weapon by the current or recent partner. Finally, exposure to sexual violence was screened when a mother answered “Yes” to any of the following questions: If the husband or partner ever physically forced the respondent to have sexual intercourse, forced the wife to have sexual intercourse despite declining it, and was forced to perform any sexual activity against her will.

#### Explanatory variables

Explanatory variables that were identified in previous studies in SSA were considered probable predictors of IPV against women in the current study [3]. These included the mother’s age, education, religion, number and gender of children, whether the woman’s pregnancy was planned, whether the respondent had a confidant, and the partner’s health risk factors. The mother’s age was categorized as 16–24, 25–34, and 35–49. Education level was based on Malawi education standards and categorized as no education, primary education, secondary education, and tertiary education. Maternal religion was categorized as Presbyterian, Catholic, Pentecostal or no religion. Number of children was coded as one, two, three, four, and five or more. The binary questions such as family planning and whether the mother had a confidant were coded yes/no.

Questions about the husband’s health risk taking behavior such as alcohol consumption, smoking and extra marital affairs were examined. The family type was grouped as monogamous and polygamous with focus on polygyny. Monogamous family refers to a type of a family whereby a man has only one wife while polygyny refers to a family arrangement in which a man marries multiple women at one time [27]. Literature has consistently shown that there is a positive relationship between polygyny and IPV in SSA region [28–30].

Household poverty was measured using the international fixed poverty line that uses purchasing power parity conversion factors of US$1.90 per day [31]. Households that were not able to spend US $1.90/day on individual household needs were counted as living below the poverty level, those who spent that amount, or more were counted as above the poverty level. The age difference of the partners were also considered and was categorized as about the same age, husband is 5 years older than wife, husband is 5 years younger than wife, husband is 6–10 years older than wife, and husband is more than 10 years older than wife. Ethnicity of respondents was also included and grouped as Chewa, Tumbuka, Ngoni, and Yawo.

Household level factors such as food security, and time taken for the mother to fetch water were also included. Household food security was measured by a Household Food Insecurity Access Scale (HFIAS). Food secure household was coded = 0, and food insecure household was coded = 1. The four HFIAS (food secure, and mildly food insecure) were coalesced to food secure, and (moderately, and severely food insecure) to food insecure households [32]. Household food access was measured by Household Dietary Diversity Scale (HDDS). Low dietary diversity households were coded = 0. This was screened when the mother affirmed that household members consumed ≤4 food groups in the past 24 h, and as 1 = minimum dietary diversity, when household members had consumed ≥5 food groups in the past 24 h [33]. Household source of domestic water was inquired and was coded 1 = borehole (protected water), and 0 = river/wells (unprotected water). Time that the mother took to fetch a pail of water was captured and coded as 0 = < 30 min, and 1 = ≥ 30 min. The detailed questionnaire has been documented and can be accessed online [34].

#### Survey administration

The survey was administered using android tablets. The tablets were loaded with the digital version of the survey using *ODK Collect. ODK Collect* is an open source android application used to administer surveys that can then collect and organize the survey data. This application allows for immediate data validation in the field. The study was administered by nine female Health Surveillance Assistants (HAS) who were trained in using the WHO protocol for conducting studies of IPV [34, 35]. The research training and pretesting of the survey questionnaire took five days. Trainers included a medical doctor, a clinical officer, and the first author who was a PhD Social Work candidate, all of whom had expertise in child and maternal health and domestic violence.

Due to the sensitivity of the research topic, the questionnaire was administered in a private consultation room at the outreach clinic during the regular mother-child clinic visit, ensuring that others present were not aware that she was participating in a research study [36, 37]. In the private consultation room, the interviewer asked mothers questions and the responses were entered in the tablet by the interviewers. This was done without the presence of other health workers and postnatal clients. This protocol was designed to maximize the privacy and safety of respondents. In four cases, the consultation room was deemed not private due to interruptions. In these cases, the interviewer agreed with the respondent on a neutral venue that was safe for both. The average duration of the interviews was 63 min with a minimum of 56 min and a maximum of two hours. The interviews were conducted in a local language Chichewa that was juxtaposed with English in ODK.

#### Research ethics review

Ethics approval to conduct this study was obtained from the McGill University Research Ethics Board in Canada (REB File #: 503–0518), and University of Livingstonia research committee in Malawi (UNILIA-REC-4/18). Written permission was also obtained from the authorities at Dowa district commissioner’s office, Dowa district health office and Mvera clinic. Oral permission were obtained from the research participants based on the advice of the research stakeholders and prior studies with other vulnerable population in Malawi (44). The rationale was that a written consent form could be easily seen by partners due to limited privacy in most households that could potentially put the mother at risk of abuse. The two ethics boards agreed with the arrangement prior to the implementation of the study project.

#### Data analysis

A total of 538 systematically selected mothers with under-five children were interviewed. There were no missing data since the questionnaire was designed so that the interviewer could not scroll to the next page in the android tablet until completing the question. Cronbach’s α was used to assess the internal reliability of the items used to determine maternal exposure to each of the four forms of IPV. In line with the WHO questionnaire, controlling behavior had five items, psychological abuse had four items, physical abuse had six items, and sexual abuse had three items (see Table 2). An α level of 0.70 or higher was considered to be satisfactory [38]. The calculated Cronbach’s α for controlling behavior was 0.81, psychological violence was 0.75, physical violence was 0.83, and sexual violence was 0.87.

Descriptive statistics were used to generate frequency tables of socio-demographic factors for mothers, children, and fathers. Univariate logistic regressions were performed to determine significant risk factors of mothers’ exposure to IPV from the selected independent variables. Four separate multivariable logistic regression analyses were performed to explore predictors of controlling behavior, emotional violence, physical violence, and sexual abuse. The variables that were significant in univariate tests were entered in the multivariate logistic regression models using forward method.

Multicollinearity of independent variables was tested and a variance inflation factor (*VIF*) of 2.314 was obtained, demonstrating that the tested independent variables were not similar and the regression coefficients estimates were reliable [39]. A fixed effects model was used to account for the intracluster homogeneity effect of the 6 study locations in the analysis [40]. The results of each of the multivariate analyses with 95% confidence interval (CI), including both crude (CORs) and adjusted odds ratios (AORs), are reported in Table 3. A *p* value of less than 0.05 was considered statistically significant in the study. The data were analysed using an IBM Statistical Package of Social Sciences (SPSS) for Windows version 23.0 (IBM Corp., Armonk, NY, USA).

## Results

### Descriptive statistics

A total of 538 mothers aged 16 to 49 years with children under five years of age consented to participate in the study. The description of the sample is presented in Table 1. The mean age of mothers was 27.64 years (SD = 7), and the reported husbands’ mean age was 32.46 years (SD = 8.23), while the mean age of children was 21.58 months (*SD* = 14.4 months). Over half of the mothers (51%) were members of CCAP, 12% were Roman Catholic, and 37% belonged to Pentecostal churches. A total of 168 mothers (31%) had only one child, 121 (23%) had two children, 106 (20%) had three children, 61 (11%) had four children, and 79 (15%) had five or more children.

**Table 1:** Socio-demographic characteristics of mothers, their partners, and children

Variable	Age (years)	***N*** = 538	Percentage
Mothers’ characteristics	15–24	224	41.6
	25–34	209	38.8
	35–49	105	19.5
	Education		
	No education	81	15.1
	Primary	370	68.8
	Secondary	87	16.2
	Religion		
	CCAP	275	51.1
	Catholic	63	11.7
	Pentecostal churches	200	37.2
	Ethnic background		
	Chewa	524	97
	Other tribes	14	3
	Has no confidant	144	26.8
	Age comparison with partner		
	About the same age	290	53.9
	Partner five years older	152	28.4
	Partner five years Younger	5	0.9
	Partner 6–10 Years older	57	10.6
Children’s characteristics	Sex		
	Female	263	48.9
	Male	275	51.1
	Age range		
	1–5	72	13.4
	6–11	90	16.7
	12–23	156	29
	24–59	220	40.9
Husbands’ characteristics	Age category		
	15–24	87	16.2
	25–34	245	45.5
	35–49	206	38.3
	Educational level		
	No education	77	14.3
	Primary	303	56.3
	Secondary	154	28.7
	Smoke	138	26.0
	Polygamous	119	22
	Drink alcohol	230	43
	Infidelity	161	30
Household characteristics	Poverty level (US$ 1.90/day)		
	Below poverty line	515	95.7
	Above poverty line	23	4.3
	Food insecure	320	59.5
	Time taken to draw water (wife)		
	<30 min	314	58
	≥ 30 min	224	42
	Number of children		
	One	168	31.2
	Two	121	22.5
	Three	106	19.8
	Four	61	11.3
	Five or more	79	14.7

In terms of education it was calculated that 370 (69%) mothers had primary school education, 87 (16%) had a secondary education, and 81 (15%) had no formal education. It was also found that 87 male partners (16.2%) were aged between 15 and 24 years, 245 (45.5%) were age 25–34 years, and 206 (38.3%) were between 35 and 49 years. It was also found that 230 male partners, (43%) consumed alcohol, 119 (22%) had a polygyny family, 161 (30%) had extra marital affairs, and 138 (26%) were smokers. Results suggested that 314 households did not have ready access to portable water, 320 were food insecure, and at least 95% of the households lived below the poverty line.

### Prevalence of IPV against mothers of children under five

The prevalence of IPV against mothers is presented in Table 2. Overall prevalence of all four forms of IPV against mothers of under-five children was 60.2%. The prevalence of controlling behavior was 74.7%. It was found that 60.6% of the sampled mothers had partners who did not allow them to talk to other men, and 49% reported that their partners had accused them of being unfaithful. Just over 50% of mothers were not permitted to socialize with their friends, while 40% were discouraged from being in contact with their relatives. It was also found that 53.7% of mothers were being stalked by their partners.

**Table 2:** Prevalence of intimate partner violence against mothers of under-five children in Dowa rural, 2018

Forms of violence	***N*** = 538	Percentage
**Controlling behavior**		
Ever jealous or angry if you talk to a male person	326	(60.6)
Frequently accuses you of being unfaithful	264	(49.1)
Does not permit you to meet your female friends	270	(50.2)
Tries to limit your contact with your family	220	(40.9)
Insists on knowing where you are	289	(537)
Summary measure of controlling behavior	402	(74.7)
**Psychological abuse**		
Ever insulted you or made you feel bad	207	(38.5)
Humiliated you in front of others	191	(35.5)
Threatened to hurt you or someone you care	156	(29.0)
Did things to scare or intimidate you purposively	150	(28.0)
Summary measure of psychological violence	266	(49.4)
**Physical violence**		
Slapped	176	(32.7)
Pushed	149	(27.7)
Hit/punch	88	(16.4)
Kicked/dragged/beaten	134	(24.9)
Choked or burned you on purpose	37	(6.9)
Threatened or attacked you with weapon	39	(7.2)
Summary measure of physical violence	235	(43.7)
**Sexual violence**		
Pressured you to have sex through harassment, threats or tricks and succeeded	350	(65.0)
Physically forced you to have sexual intercourse with him when you did not want to	253	(47.0)
Force you with threats or in any other way to perform sexual acts you did not want to	230	(42.8)
Summary measure of sexual violence	394	(73.2)

In addition, 182 mothers (33%) reported experiencing at least one form of IPV within their first year of marriage, while 226 mothers (41%) reported that their partners started the abuse after they had been married between two and five years. Finally, 91 mothers (17%) indicated that their partners became abusive after they had been married six or more years.

The prevalence of psychological abuse against mothers of under-five children was 49.4%, with 38.5% reporting having been insulted by their partners and 35.5% were humiliated in public. In addition, 29% of mothers felt threatened by their partners’ anger, while 28% reported that their partners had scared or intimidated them on purpose. The prevalence of physical IPV against mothers was 43.7%, including 32.7% of mothers who had been slapped, 27.7% who had been pushed and 16.4% who had been punched. In addition, 24.9% of mothers were kicked or dragged or beaten, 6.9% were choked or burnt on purpose, and 7.2% were threatened or attacked using a weapon.

Finally, the prevalence of sexual IPV by the current or most recent partner was 73.2%. Most women (65%) reported that their partners forced them to have sex through pressure, threats, harassment or tricks. Nearly half of the mothers reported that their partners had physically forced them to have sex without their consent. In addition, 42.8% of mothers reported that their partners forced them to perform sexual acts against their will. The study also indicates that 47.8, 38.5, and 24.2% of mothers reported that they experienced psychological abuse, physical, and sexual violence respectively within 12 months prior to this research. In total, 164 mothers (30.5%) reported having experienced all four forms of violence throughout the duration of their engagement and marriage.

### Predictors of IPV against mothers of children less than five years

The results of logistic regressions are presented in Table 3. The Crude ORs suggested that male partner behaviors and characteristics, such as alcohol consumption, smoking, polygyny, previous divorce, and age, were significant predictors of IPV against mothers. Having a confidant was the mothers’ only characteristic that predicted less exposure to IPV. At the household level, a mother who spent more than 30 min fetching water was at risk of experiencing IPV. None of the children’s characteristics were significant predictors of IPV against mothers. Likewise, the mothers’ characteristics, including education, religious affiliation, age, membership in a village bank, and ethnic background, were not associated with their exposure to violence. Household characteristics, such as number of children, food security, poverty level, and farm size, were not significant predictors of any form of IPV against mothers.

**Table 3:** Crude and adjusted odds ratios (95% CI) for factors associated with IPV among mothers of under-five children

Variables	Model 1 Controlling behavior		Model 2 Psychological violence		Model 3 Physical violence		Model 4 Sexual violence	
	Crude OR	AOR	Crude OR	AOR	Crude OR	AOR	Crude OR	AOR
*Partner drinks beer*								
No	1	1	1	1	1	1	1	1
Yes	1.73***	1.07	1.62***	1.16	1.52**	1.18	1.99***	2.0***
*Partner smoke*								
No	1	1	1	1	1	1	1	1
Yes	2.39***	1.91*	1.78***	1.17	1.78***	1.06	1.75*	1.07
*Marriage type*								
Monogamous	1	1	1	1	1	1	1	1
Polygamous	2.17***	1.56	1.83***	1.72	2.1***	1.06	1.32	1.31
*Infidelity partner*								
No	1	1	1	1	1	1	1	1
Yes	5.39***	4.97***	2.4***	2.14***	2.69***	2.29***	1.86***	1.49
*Divorced before*								
No	1	1	1	1	1	1	1	1
Yes	1.76*	1.81	1.86	1.64	1.71***	1.37	1.22	1.31
*Age of partner*								
15–24	1	1	1	1	1	1	1	1
25–34	0.58*	0.77	0.54*	0.70	0.52**	0.74	0.74	1.08
35–49	0.98	1.08	0.74	0.80	0.63**	0.66	0.61	0.64
*Time to fetch water*								
30 Minutes or less	1	1	1	1	1	1	1	1
More than 30 min	2.44***	2.03***	2.44***	1.45	1.85***	1.59**	2.61***	2.27***
*Mother has confidant*								
No	1	1	1	1	1	1	1	1
Yes	1.13	1.09	1.03	1.03	0.67*	0.69	1.13	1.18

*P <.05. **p <.01. ***p <.001

Using multivariate regression analyses, all the explanatory variables that were significant predictors of IPV at the univariate level were examined. The results of the multivariate logistic regression of controlling behaviors (Table 3, Model 1) showed that mothers whose partners had extra marital affairs were more likely to experience controlling behavior (AOR: 4.97, 95% CI: 2.59–8.55, *P* = 0.001) than mothers whose partners were not involved in extra marital affairs.

The odds of experiencing controlling behavior was higher among mothers with partners who smoked tobacco (AOR: 1.91, 95% CI: 1.59–2.55, *P* = 0.05) than among mothers whose partners did not. Mothers who spent more than 30 min each day fetching a single tin of water were at greater risk of experiencing IPV controlling behavior (AOR: 2.03, 95% CI: 1.023–2.640, *P* = 0.001) than mothers who spent less than 30 min fetching water. Although women married to partners who consumed alcohol, smoked tobacco, and had been divorced were at increased risk of experiencing controlling behaviors in bivariate analyses, these results were not significant in the multivariate analyses.

For psychological violence, the results of multivariate analysis (Table 3 model 2) demonstrated that mothers who reported that their partners had extra marital affairs were 2.14 times more likely to experience psychological violence (AOR: 2.14, 95% CI: 1.486–3.472, *P* = 0.001) than mothers whose partners had no extra marital affairs. For physical abuse, the multivariate analyses (Table 3, Model 3) indicate that mothers whose partners had extra marital affairs were at an increased risk for experiencing physical violence (AOR: 2.29, 95% CI: 1.48–3.94, *P* = 0.001) than mothers whose partners did not have extra marital affairs. Mothers who spent more than 30 min fetching water were more likely (AOR: 1.59, 95% CI: 1.02–2.32, *P* < 0.01) to experience physical IPV than mothers who spent less than 30 min.

Regarding predictors of sexual IPV (Table 3, Model 4), the multivariate analyses showed that mothers married to husbands who consumed alcohol were more likely to experience sexual violence (AOR: 2.00, 95% CI: 1.17–3.41, P = 0.001) than mothers whose husbands abstained from alcohol. In addition, mothers who spent 30 min or more fetching water were at an increased risk of experiencing sexual IPV (AOR: 2.27, 95% CI: 1.33–3.39, *P* = 0.01) than mothers who spent less time on this task.

## Discussion

The study described in this article examined the prevalence and determinants of IPV against mothers of under-five children in order to contribute to the AWD goal of accelerating gender equality and empowerment of women. It also provide a context for understanding of various social determinants of poor maternal and child health outcomes in Dowa district. The average prevalence of all four forms of IPV against mothers of under-five year old children was at 60.2%. The study suggest that in Malawi, mothers of under-five children are vulnerable population to IPV perpetrated by the recent partner as compared to the women in the general population. The findings support a recent study in Lilongwe district, Malawi that found that the burden of IPV against girls and young women (15–24 years) were extremely high (75%) [41]. It is important to note that Lilongwe is neighboring district of Dowa where this study was conducted. These two case studies suggest that national data of IPV against women in the general population underrepresent the burden of IPV in specific population of women and girls in central Malawi.

The prevalence of controlling behavior (74.7%) found in this study was significantly higher than the prevalence of controlling IPV in the general population of women (24%) in Malawi found in previous studies [4]. There are several possible explanations for the higher prevalence rate in the current study. First, the nationally representative studies of IPV sampled women between 15 to 49 years, including women who had never married and those who were divorced or widowed [4], while the sample of this study was more homogeneous, consisting of married women from the rural areas of one district of Malawi. In addition, since mothers were asked about their relationship with current or most recent partner, it is possible that they were better able to recall specific incidents and behaviors.

Further, this study was administered by female community health workers who have served in the area in the range of nine to twenty years providing primary health care services, such as family planning, antenatal, and postnatal services. Therefore, it is possible that mothers would be more open with these community health workers, with whom many had developed personal relationships and trust over time.

The prevalence of psychological IPV in the current study was 38.5%, slightly higher than the findings of the nationally representative study of IPV against women, which found a psychological IPV prevalence of 30% [4]. However, the findings were lower than those in the systematic review of studies of IPV in Ethiopia [42], where 51.7% of women were found to have experienced psychological abuse. On the other hand, a study conducted among pregnant women in Rwanda found that 20.6% of participants had been victims of psychological abuse [43]. Although the prevalence of psychological IPV in Dowa is within the range of many studies in SSA and lower than a study in Lilongwe district in Malawi [41], it will be important to continue to explore regional and methodological differences to better understand these variations in prevalence among studies.

The current study found that 43.7% of mothers experienced physical violence by their current or most recent partner. This finding is slightly higher than the average prevalence of physical IPV found among the general population of women in SSA [44]. However, studies in Guinea, Tanzania, and Gabon measured prevalence as 54, 53, and 46% respectively, higher than the present findings [45, 46]. The most common forms of physical IPV against mothers in this study were slapping (32%) and pushing (28%). This is in line with another study in SSA where women reported being slapped (23%) and punched (11.2%) by their partners [47].

In this study, the prevalence of sexual IPV (73.2%) was significantly higher than the average prevalence of sexual IPV found in other studies of SSA (13.3%). The prevalence in the current study was also significantly higher compared to studies conducted in Ghana (30%), Uganda (27%), and the Democratic Republic of Congo (26%), which are considered high in the SSA region [1]. The difference between this results and those of other studies may be explained by the sample selection. The current study specifically sampled homogenous mothers who were in conjugal relationships or were recently broke up with their partners and thus assumed to be sexually active. Culturally in Malawi, once a woman is married, she is generally expected to be submissive to her husband, particularly with regard to sex [48]. Therefore, the high prevalence of sexual abuse in this study may reflect cultural beliefs that support sexual aggression by men and submission by women. It is interesting, however, that the women reported that their partners’ behavior was in violation of their wishes and welfare, an indication that all wives do not fully accept these cultural norms, a finding which deserves further exploration in this study area [1].

In this study, women with partners who had extra marital sexual affairs were at greater risk for experiencing controlling, psychological, and physical violence than women who reported that their partners did not had extra marital affairs. This finding is consistent with the literature in SSA that found that marital infidelity by men was a significant trigger of IPV against women [1, 49]. Studies have shown that when women confront their partners after discovering their infidelity, some partners respond aggressively, which can escalate into emotional or physical violence [50].

This study supports previous research in SSA that suggests that alcohol consumption by male partners increases the odds of women experiencing IPV [51, 52]. Recent studies have shown that, biologically, alcohol diminishes the judgment and perception of the drinker, and thus partners who are under the influence of alcohol are more likely to lose self-control and engage in violent behavior [51].

This study also found that mothers who were spending 30 min or more drawing water had a greater chance of experiencing controlling, physical, and sexual IPV. This finding is in line with a qualitative study conducted in Bangladesh where women who fetched water from distant wells were more likely to experience IPV [29]. In Malawi, mothers are expected to fulfill multiple household roles such as preparing food and taking care of children [53, 54]. Since fetching water from distant wells takes time away from other tasks, the IPV may be interpreted within the village context as a male partner’s right to reprimand the wife for not fulfilling marital obligations. The issue of cultural norms with regard to IPV is one that deserves further attention in the context of mothers nursing under-five children [55].

Finally, it was found that mothers who were married to tobacco smokers had a higher risk of experiencing controlling IPV than mothers who were not married to smokers. The findings are consistent with the results of a study in Bangladesh that found tobacco smoking among men linked to perpetration of IPV [56]. Similarly, in two nationally representative studies conducted in the United States, tobacco smoking was a significant mediating factor for men’s perpetration of IPV [57, 58]. One potential explanation for the observed link between IPV against women and tobacco smoking by partners can be drawn from biology literature. Research has found that the nicotine in tobacco can undermine efforts to regulate smokers’ emotions such as anger, hostility, impulsivity, and anxiety. Therefore, partners who were under the influence of nicotine could be more likely to show aggressive behavior than partners that were not under the influence of nicotine. Following the ecological model, there may well be additional cultural and social factors influencing the relationship between smoking and IPV. Regardless, further research is needed to understand the relationship between IPV, and smoking found in this study.

### Implications for practice

There are several recommendations that emerged from this study. First, it is suggested that programs that aim at mitigating IPV against mothers in Dowa district should also take into account macro-level social work and public health practices. In particular, the programs should also consider taking a prevention approach by developing interventions that address husbands’ risk factors. In fact, the primary focus should be on transformative hegemonic community gender-norms that can motivate husbands to change their social risk behaviors, particularly infidelity, polygyny, alcohol consumption, and tobacco smoking.

It is also recommend that international, national, and district public health policy should prioritize potable water development projects in the study area. Such projects will not only improve access to safe water for families with young children, but the current study suggest that it may also reduce the likelihood of IPV against mothers. It has been found that time spent by mothers fetching water outside the home may restrict other equally important responsibilities, such as childcare. It is believed that mothers’ time poverty triggered IPV. Caution should be taken from this recommendation, as the study is not encouraging partners in this study area from secluding mothers from their social networks on pretense of enforcing social norms. Such act is a violation of women’s right to freedom of movement and is punishable by law.

### Strengths and limitations

This is the first local study to collect data on the prevalence and determinants of IPV against mothers of children under five years of age in rural areas of Dowa district in Malawi. A primary strength of the study is the use of the well-established WHO multi-country questionnaire previously used and validated in Malawi. Thus, the results of this study can be compared to those of other studies that use the same instrument.

However, this study also had limitations. Since this is a cross-sectional study, it cannot establish a causal relationship between IPV against mothers and the risk factors associated with it. Further, despite conducting a culturally relevant mixed-methods approach, it may be likely that mothers under-reported their experiences of IPV for several reasons.

First, as a retrospective study, mothers were asked to recall situations and behaviors that they had experienced in the past, and it is possible that recall bias could influence results, particularly the memory of incidents of IPV. Second, despite increased awareness regarding the detrimental effects of IPV against women in Dowa district, including laws in place intended to protect those experiencing it, IPV remains a sensitive issue. Due to legal repercussions for perpetrators, some respondents may have been afraid to disclose that they were abused in order not to bring harm to their husbands or to lose their husband’s income and other support roles if they are convicted of IPV and imprisoned or fined. Although a higher percentage of mothers in Dowa district reported experiencing IPV in this study than previous studies in Malawi and SSA, it is possible that IPV against mothers in the district continues to be under-reported by mothers for larger cultural, social, and economic reasons.

As a crucial factor in the rights, dignity, and well-being of women in Dowa district, this study suggest that IPV should be addressed at both micro and macro levels. For instance, there is a need to strengthen programs that promote direct IPV protection services such as policing and legal registrations. In addition, the risk behaviors engaged in by husbands identified by this study should be addressed through social programming such as participatory community discussion groups. Finally, there should be coordination between IPV prevention and public health policy, since the study suggests that potable water projects not only improve the health of the public but may also reduce the risk of IPV for mothers with young children.

## Conclusion

This study has revealed that there are still challenges to attain the African Union Women Decade goals of ensuring zero tolerance to gender based violence and empowerment of women in remote areas of Dowa district in Malawi. It was observed that an average of 60.2% of mothers with under-five children experienced all four forms of IPV. In the study, most of the risk factors of IPV against mothers were found to be related to husbands’ health risk behaviors. These included alcohol consumption, tobacco smoking, polygyny, and infidelity. Women who spent more time fetching water were also more likely to experience violence perpetrated by their partners. Community-based participatory intervention models are proposed to mitigate IPV against women. In particular, government and its development partners are encouraged to involve local stakeholders to find relevant and culturally sensitive solutions to the problem of IPV against mothers of under-five children.

## Data Availability

The study involved capturing of sensitive data according to WHO standards. We documented mothers’ disclosure of violence by their current husbands. Due to the sensitivity of the study McGill REB did not recommend sharing the raw data publicly. In case some scholars may need the raw data for further analysis, they can contact the corresponding author for appropriate ethical steps before accessing the data.

## Abbreviations

AOR: Adjusted odds ratios
CI: Confidence interval
IPV: Intimate partner violence
NSO: National Statistics office
SSA: Sub Sahara Africa
US$: United States dollar
WHO: World Health Organization
